# New Diagnostic and Therapeutic Approaches for the Care of the Severely Injured Patient

**DOI:** 10.3390/jcm9113468

**Published:** 2020-10-28

**Authors:** Frank Hildebrand, Klemens Horst

**Affiliations:** Department of Trauma and Reconstructive Surgery, University Hospital RWTH Aachen, 52074 Aachen, Germany; fhildebrand@ukaachen.de

Severe trauma remains a leading cause of death, especially in the younger population. Pre- and intrahospital diagnostic and therapeutic procedures according to well-established guidelines (e.g., Advanced Trauma Life Support^®^ (ATLS^®^, American College of Surgeons, The Committee on Trauma, Chicago, IL, USA) are widely accepted to beneficially influence posttraumatic outcomes. However, the further assessment and treatment of severely injured patients still remain challenging due to a high variety of injury patterns and manifold patient-specific factors. The latter individual aspects (e.g., age, gender) have been shown to modulate the posttraumatic immune response, which in turn has a significant impact on the clinical course after severe trauma. To improve posttraumatic outcomes, further clinical and experimental research is needed to better understand the underlying pathomechanisms and to improve diagnostic and therapeutic strategies. This Special Issue of the Journal of Clinical Medicine therefore focuses on recent aspects of the management of severely injured patients. Based on data from clinical studies, pathomechanisms, new diagnostic and therapeutic approaches, as well as outcome observations are presented.

The ABCDE scheme represents the basis of the ATLS^®^-recommendations of the American College of Surgeons (ACS) Committee on Trauma. According to this algorithm, securing the airway (A-problem) and ensuring ventilation (B-problem) are the first priorities when approaching a severely injured patient. However, the establishment of a safe airway might be very difficult. In a monocentric, prospective study on a physician-staffed air ambulance, Macke et al. proved that C-MAC (Storz Medical, Tuttlingen, Germany) videolaryngoscopy in the prehospital setting was associated with significantly better first pass success for endotracheal intubation compared to direct laryngoscopy [1]. As lethal hemorrhage remains a leading cause of early death in severely injured patients, the maintenance or restoration of a hemodynamically stable situation (C-problem) is another priority of the ATLS concept. To estimate the risk of massive transfusion, Horst et al. introduced the modified Trauma-Induced Coagulopathy Clinical Score (mTICCS) as a new scoring system to identify patients in need of massive transfusion. Based on an analysis of a trauma registry, the authors successfully validated mTICCS against well-established and more sophisticated algorithms and found that it presents an useful tool to predict the need for massive transfusion early [2]. Focusing on neurological aspects (D-problem), Popp et al. validated a CT protocol with mandatory dedicated computer tomography angiography (CT-A) to detect cervical artery dissections in severely injured patients [3]. From the results of their monocentric, prospective study, the authors concluded that their imaging pathway is likely to reduce possible therapeutic delays. Peripheral neurological symptoms are mainly caused by injuries of the spinal column. Based on a retrospective analysis, Kobbe et al. support the concept of early spinal stabilization in severely injured patients with AOSpine B-/C-type injuries, especially of the thoracic spine. However, in AOSpine A-type injuries, the beneficial impact of early spinal stabilization seems to be overemphasized and the benefit should be weighed against the risk of patients’ deterioration during early spinal stabilization [4].

Chest trauma is one of the most common injury types after severe trauma and also has a significant impact on the clinical course. A flail chest is a serious condition after chest trauma, however the optimal treatment (conservative vs. operative) for this kind of injury is not clarified. In their retrospective study, Niemann et al. did not find differences in clinical parameters (e.g., the duration of mechanical ventilation, analgesia requirements, pneumonia incidence) between conservatively and operatively treated patients. However, a prolongation of both the length of intensive care unit (ICU) stay and hospital treatment duration was observed in operatively treated patients [5]. In a further study with a retrospective design, Eckhardt et al. confirmed the relevance of chest trauma in the posttraumatic course (longer ventilation time, prolonged ICU stay, increased mortality) Interestingly, being overweight resulted in an independent mortality reduction in patients with severe chest trauma [6]. It was postulated that overweight-associated factors, such as nutritive advantages or a modulated posttraumatic immune response, might be responsible for the observed survival benefit [6].

Immunological alterations after severe trauma were also discussed by two other manuscripts of this Special Issue. In a clinical review, Kobbe et al. summarized the current knowledge about the neuroendocrine modulation of the immune system during trauma and sepsis and the potential arising therapeutic options [7,8]. Schindler et al. retrospectively investigated the relevance of traumatic brain injury (TBI) with or without concomitant injuries on systemic inflammation and other markers of neurological damage (e.g., S100b). They found that TBI is associated with a decreased interleukin expression. However, the diagnostic relevance of potential markers of neuronal damage seems to be limited [7,8]. Two other studies performed blood analyses in order to investigate posttraumatic changes in the humeral and cellular inflammatory response as well as in the complement and coagulation system [9,10]. In this context, Sturm et al. focused on the CD4^+^ T lymphocyte population after severe trauma. They observed that the number of T-regulatory cells was decreased within this population; however, their inductiveness (e.g., by IL-10) was increased [9,10]. Vollrath et al. described a promising predictive value of C5a and thrombin-activatable fibrinolysis inhibitor (TAFI) levels for the development of sepsis [9,10]. Zechendorf et al. investigated ribonuclease (RNase) 1 and its antagonist (RNase inhibitor (RNH) 1 to determine the association of post-operative acute kidney injury (AKI) and in-hospital mortality [11]. RNase 1 belongs to the group of antimicrobial peptides that is elevated in septic patients and indicates the prediction of two or more organ failures. Both RNase1 and RNH1 may be therapeutically relevant and may represent biomarkers for post-operative AKI and in-hospital mortality [11]. Hesselink et al. concentrated on infections after trauma and described persistent inflammation, immunosuppression, and catabolism syndrome (PICS) as a potential explanation of recurrent infections. PICS was associated with prolonged hospitalization, many surgical procedures, and frequent readmissions. Therefore, PICS forms a substantial burden on the patient and the hospital, despite its low incidence [12].

Diverse studies of this Special Issue focused on severe trauma in the elderly population, which clearly underlines the increasing clinical importance of this topic. Stassen et al. found a high prevalence of sarcopenia among severely injured patients that was particularly pronounced in the older patients. As sarcopenia was also identified as a predictor of 30-day mortality, the clinical relevance of the identification of a low muscle mass via CT scan seems to be obvious [13]. Focusing on fragility fractures of the pelvis (FFP), Rommens et al. retrospectively found that even isolated fractures of the anterior pelvic ring without any involvement of the posterior ring (FFP Type I) were associated with relevant mortality, a loss of independence, restricted mobility, and a decreased quality of life. The authors concluded that pubic ramus fractures are indicators of the need to optimize the patient’s general condition [14]. In fractures affecting the posterior pelvic ring, Hartensuer et al. explored in a retrospective study the safety, effect, and feasibility of percutaneous sacroiliac (SI) screws with and without augmentation. Cement augmentation seemed to reduce the complication risk in FFP patients and shorten their hospital stay, without increased specific complications or correlated neurological impairment [15]. In order to ensure the earliest possible mobilization after FFP, Pfeufer et al. were able to reliably detect the extremity load in patients with FFP with the help of an insole device [16]. They concluded that this technique might offer a great potential to improve the choice and time of treatment of FFP. In a study based on the “Trauma Registry DGU”, Freigang et al. clearly showed that severe trauma also significantly affects the long-term outcomes of elderly patients with no improvement in their health-related quality of life (HRQoL) up to 24 months after trauma in patients ≥65 years [17].

Apart from injury pattern and specific patient groups, the treatment of severely injured patients is associated with high personnel and structural requirements. Therefore, the targeted and reasonable use of these resources is of upmost importance. Heinrich et al. showed in a retrospective analysis that slightly injured patients (Abbreviated Injury Scale (AIS) ≤ 1, Maximum Abbreviated Injury Scale (MAIS) ≤ 1 and Injury Severity Score (ISS) ≤ 3) might not need to complete a ≥ 24 h monitoring [18]. In the light of the economic use of resources, the importance of predicting risk factors associated with specific injury pattern as well as an analysis of the need for extended diagnostic procedures are presented by two further manuscripts of this Special Issue [19,20]. In a study using data from the “Trauma Registry DGU”, Weber et al. defined the risk factors and accident mechanisms which are indicative of the presence of knee injuries and described a significant impact of this injury pattern on the clinical course [19,20]. Halvachizadeh et al. retrospectively investigated the significance of intraoperative computed tomography (CT) for the operative treatment of intra-articular fractures after severe trauma. They found that CT was associated with improved surgical results and concluded that the decrease in reoperations may justify its use, despite the higher CT-associated radiation dose [19,20].

Further studies of this Special Issue also focus on innovative therapeutic approaches. As patients with spinal cord injury (SCI) are likely to exhibit hemostasis disorders, Mackiewicz-Milewska et al. assessed the effects of different rehabilitation programs on hemostasis in patients with SCI. They found that a 4-week program was suggestive of positive results, more pronounced in the subacute and chronic state (≥3 to 6 months) rather than in the short term after SCI [21]. In severely injured patients, bony mal- or non-unions are a regularly observed complication. Wang et al. therefore investigated the possibility of an arthroscopy-assisted corrective osteotomy (AACO), reduction, internal fixation, and strut allograft augmentation for cases affecting the tibial plateau. The authors proved good radiological and clinical results [22]. Additionally scarring is a further common problem after severe injury. Joo et al. evaluated the effects of extracorporeal shock wave therapy (ESWT) on hypertrophic scars of the hand caused by burn injuries. They were able to prove that ESWT was effective in decreasing pain, suppressing hypertrophic scarring, and improving hand function [23]. In addition, the same study group showed that a virtual-reality (VR) based rehabilitation program is likely to be as effective as conventional rehabilitation programs for recovering function in a burned hand [24].

In conclusion, the presented articles reflect the current knowledge about the pathomechanisms associated with the impact of severe injury and its consequences for the further clinical course on the one hand and points out new insights in regard to diagnostic and therapeutic approaches in severely injured patients on the other hand. Furthermore, interesting aspects regarding future directions for the care of the severely injured patient are illustrated, and we hope you enjoy reading our selections of topics regarding the importance of investigating all aspects associated with severe trauma.
